# May I see what you see? Predicting visual features from neuronal activity

**DOI:** 10.1016/j.isci.2024.108819

**Published:** 2024-01-09

**Authors:** Vikram Ravindra, Chih-Hao Fang, Ananth Grama

**Affiliations:** 1 University of Cincinnati, Cincinnati, OH, USA; 2 Purdue University, West Lafayette, IN, USA

**Keywords:** Medical imaging, Systems neuroscience, Signal processing, Signal reconstruction, Machine learning

## Abstract

Understanding brain response to audiovisual stimuli is a key challenge in understanding neuronal processes. In this paper, we describe our effort aimed at reconstructing video frames from observed functional MRI images. We also demonstrate that our model can predict visual objects. Our method constructs an autoencoder model for a set of training video segments to code video streams into their corresponding latent representations. Next, we learn a mapping from the observed fMRI response to the corresponding latent video frame representation. Finally, we pass the latent vectors computed using the fMRI response through the decoder to reconstruct the predicted image. We show that the representations of video frames and those constructed from corresponding fMRI images are highly clustered, the latent representations can be used to predict objects in video frames using just the fMRI frames, and fMRI responses can be used to reconstruct the inputs to predict the presence of faces.

## Highlights

• NN architecture showing similarity in representations of visual stimuli and fMRI
• Predict objects in video frames using just the fMRI frames using the representations
• fMRI responses can be used to reconstruct faces that were present in visual stimulus

## Graphical abstract

**Figure:**
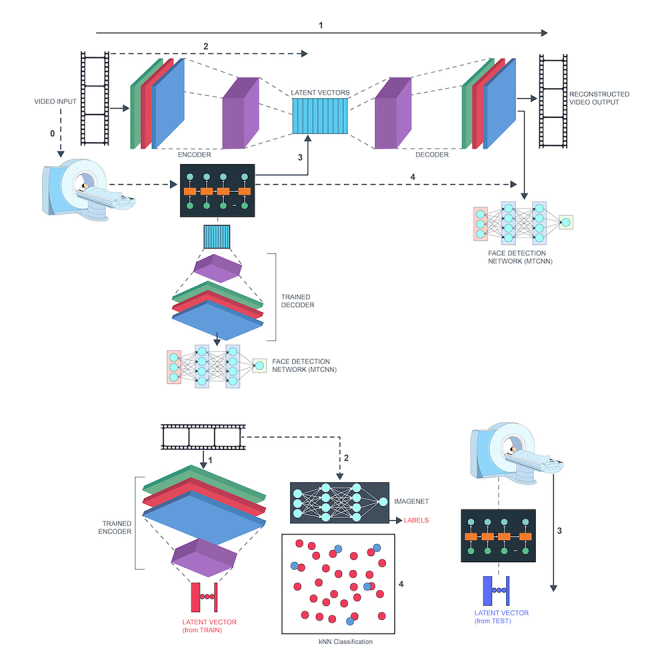

## Introduction

Functional MRIs (fMRIs) are biomedical images that have three spatial (x-y-z) dimensions, at a resolution of $\approx 1mm^{3}$, and a temporal dimension at a resolution of ≈1s. They capture dynamic brain activity at rest and while performing tasks. fMRIs are used widely in neuroscientific studies to understand complex processes such as cognition, reasoning, memory, and processing of audio, visual, and olfactory inputs. Further, fMRIs are used for diagnosis and prognosis of several neurological diseases such as Alzheimer disease,^1^ stroke,^2^ depression,^3^ schizophrenia,^4^ and bipolar disorder.^5^ fMRI studies typically characterize healthy processes by measuring temporal coherence between specific brain regions. Then, the dysregulation of these processes is used to recognize different diseases and disorders. The field of connectomics is dedicated to mapping and understanding the structure and function of the brain.

**Figure 1:**
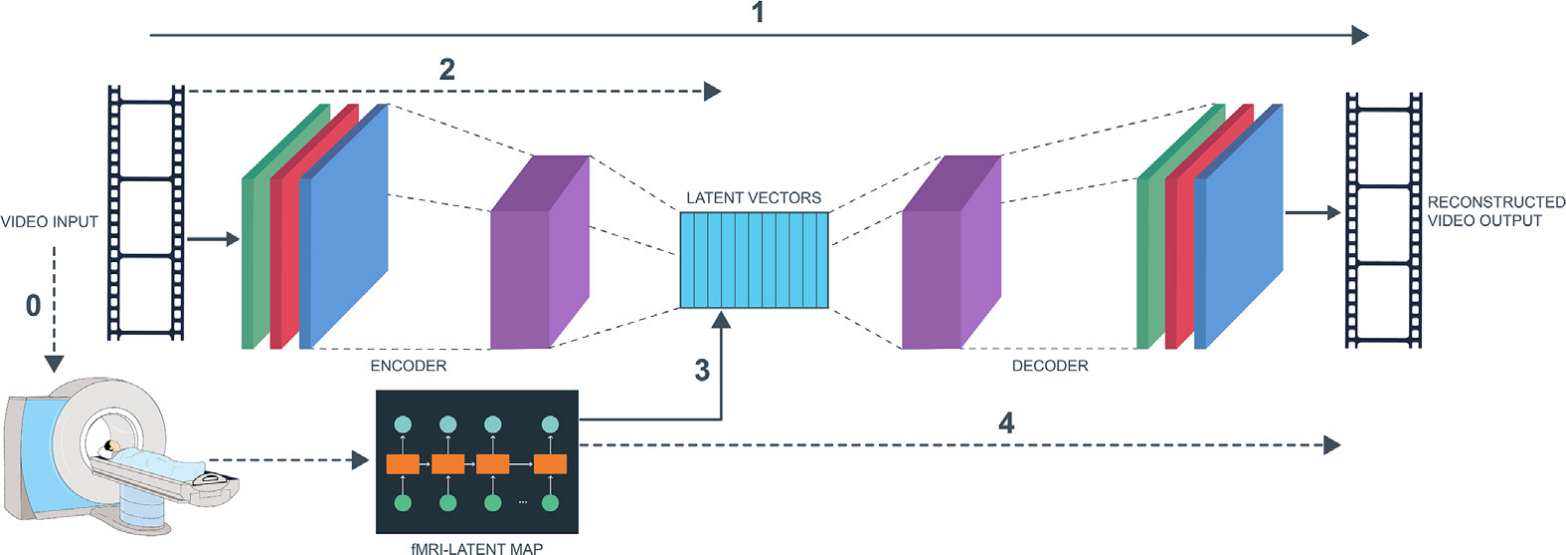
Schematic representation of our overall setup (1) we train the autoencoder neural network on the training video clips; (2) we convert video frames into latent representations; (3) we train a map from the fMRI response to latent vectors corresponding to the same visual input frames; and (4) we reconstruct video frames from fMRI using previously trained map and decoder. We note that the solid arrows correspond to training steps. Step zero is the data-acquisition stage, where a cohort of subjects watch video stimuli in an MRI machine.

**Figure 2:**
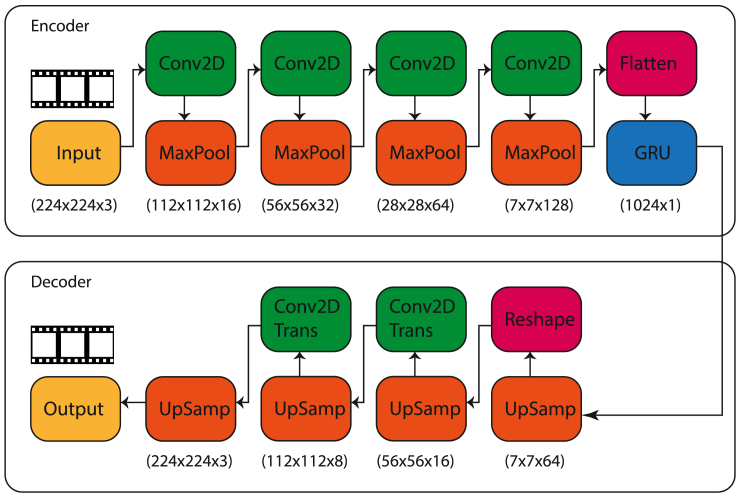
Schematic representation of our Encoder-Decoder Model: Each input frame is of dimensions 224×224×3 The encoder is a sequence of convoluted neural networks (CNNs) and pooling layers, with increasing number of channels and reduction in spatial dimensions. Finally, we flatten out the tensor and input into a Gated Recurrent Unit (GRU). The decoder is a sequence of upsampling and CNN transpose. The output of the final layer of decoder is a video frame with dimensions that match the input.

**Figure 3:**
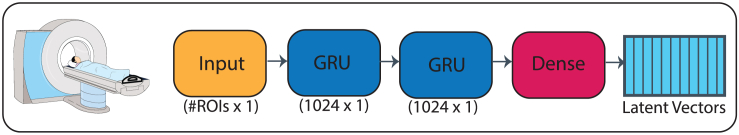
Schematic representation of the fMRI-Latent Vectors Map We have 2 layers of GRU with 1024 dimensions and tanh activation. Finally, this is followed by a linear dense layer.

In this study, we present a computational approach to construct a model that maps time-series signals from fMRIs to visual features that stimulated the observed neuroimages (Section STAR methods). Briefly, we use the architecture shown in Figure 1 on the publicly available dataset from the Human Connectome Project (HCP), which contains videos, and corresponding neuroimage responses from over 1,100 healthy young subjects (details are provided in section dataset and preprocessing). To accomplish this, we first design an encoder-decoder artificial neural network (Figures 2 and 3) for mapping input video segments to their latent representations (low-dimensional encoding) and to accurately reconstruct them (decoding).

Separately, we choose regions of interest (ROIs) from our fMRI dataset that contain neuroimage responses to the same video streams. We construct a model (a Gated Recurrent Unit [GRU] network) that maps neuronal activity in the ROIs to the latent representations of the corresponding visual stimuli. We train this model so that the latent representations best match those from the encoder acting on corresponding video frames.

**Figure 4:**
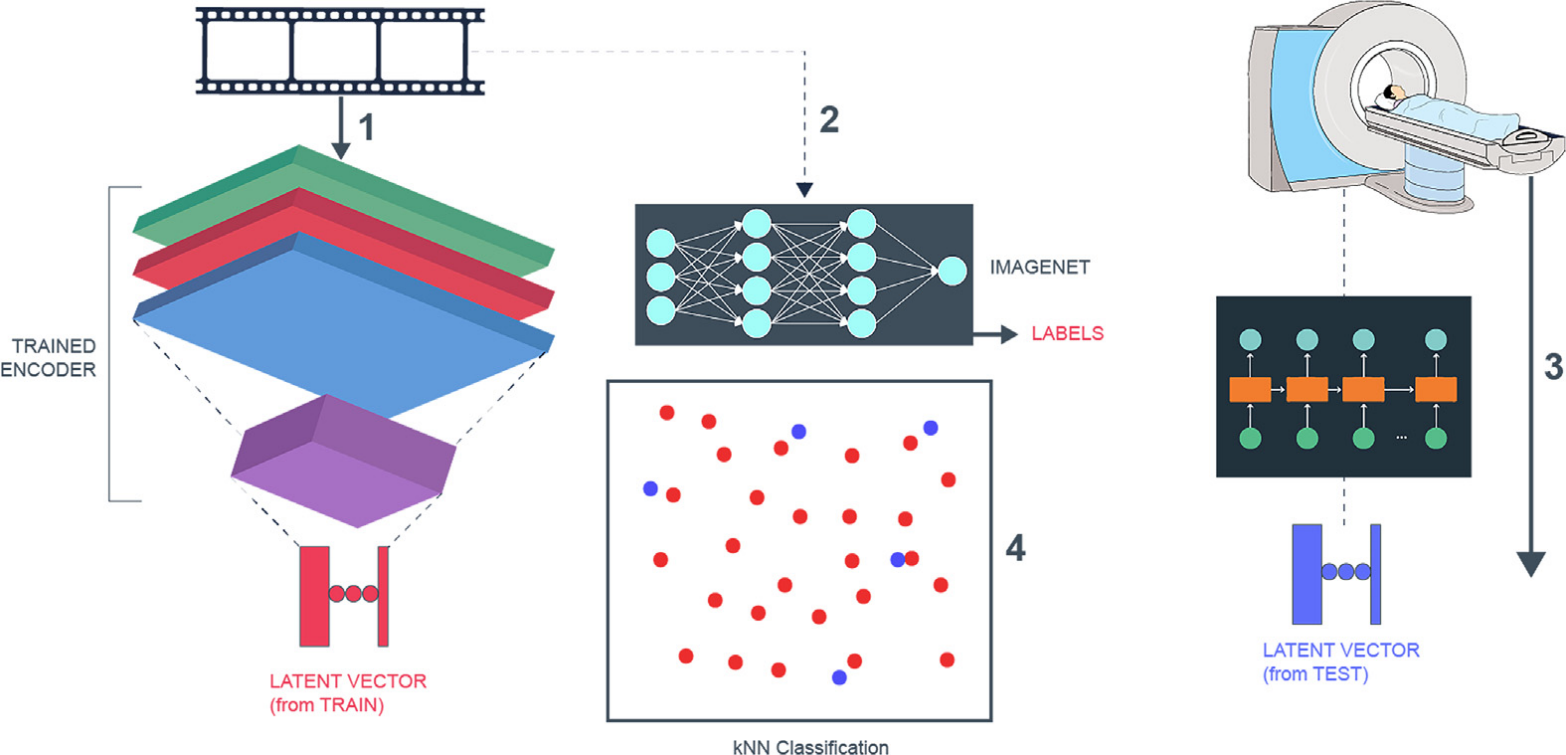
Prediction of visual features from latent space We assume that the autoencoder and the fMRI-latent map are previously trained. First, using the train video frames, we obtain the (actual) latent vectors. Then, we input the same video frames into pre-trained VGG16 on ImageNet database to classify each of the frames. Following this, we predict latent vectors by inputting test fMRI through a pretrained map. We then do k-NN classification between actual and predicted latent vectors to predict visual features in fMRI frames.

To test our models, we input unseen (test) fMRI frames into the GRU to see if the generated latent space vectors are close to those generated from the corresponding encoded videos. We also analyze the decoded versions of these two latent vectors (one from the original videos and the other from the fMRIs) to see if we can detect identical video objects in both decoded streams (Figure 4). To deal with issues of overfitting and memory in our network models, we (1) train the encoder-decoder only on the train set of video frames. This ensures that the decoder has not encountered the latent vectors corresponding to the test fMRIs or the predicted latent vectors obtained from the test fMRIs; (2) once we have trained the encoder-decoder, we fix their weights (i.e., we do not change them to optimize for fMRI inputs); and (3) we use pretrained neural networks such as VGG16 on ImageNet,^6^ WordNet,^7^ and MTCNN^8^ to understand (classify) the output images to identify objects in reconstructed images such as faces and other objects. Through these design choices, we ensure that our models and methods are robust.

## Results

We present experimental results of our image reconstruction framework on a movie-viewing dataset from the Human Connectome Project (HCP). First, we discuss the dataset and required preprocessing steps. Next, we show three key results: (1) we show that we can find a common latent feature space to express video and fMRI frames in an efficient manner; (2) we show that we can identify objects in video reconstructions from functional neuronal images; and (3) we show that we can detect the presence/absence of faces in reconstructed images consistent with video stimulus. We choose faces as illustrations of objects (and since we can use third-party networks to verify results). Our reconstructions reveal general objects in scenes. We compare our performance with that of the method proposed by Wen et al.^9^ for results (1) and (2), as theirs is the only other method that operates on naturalistic video inputs. Additionally, in result (3), we compare our approach with methods by VanRullen and Reddy^10^ and Cowen et al.^11^ as their approaches operate on faces. We provide a summary of these approaches in Section related literature. Finally, we end the section with a brief discussion on system specification.

### Dataset and preprocessing

We use the movie viewing task fMRI data collected as part of the Young Adult project of the HCP consortium, as described by van Essen et al.^12^^,^^13^ The dataset contains 7T fMRIs from 184 human subjects (male and female, left- and right-handed, age range 22–35 years). There are four sessions for each subject, each of which lasts for approximately 15 min. Each session corresponds to viewing four or five audiovisual clips, each of which is 1 to 4 min in length. The clips are separated by 20 s of rest. We note that publicly available metadata are scrubbed of all directly identifiable details such as name, contact details, etc. All HCP data and code are available for free download under GNU sharing license. An important component essential to the success of our approach is feature selection from fMRI data, making it amenable to analysis. First, we use the minimal preprocessing pipeline^14^ to rid the fMRI of spatial and temporal noise and align all images to the standardized gray coordinate system. Then, we perform global signal regression, which removes signal that is ubiquitous across all voxels. This is shown to greatly improve signal-to-noise ratio. Next, we choose a small subset of time-series from the whole-brain data, which we call regions of interest (ROIs). ROIs are chosen either on the basis of anatomy (prior knowledge) or on the basis of activity (data-driven).^15^ In our application, we define ROIs as voxels whose mean activity level during viewing is significantly different from the rest sessions between scenes. We perform a two-tailed statistical test to choose voxels that express a distinct response to visual stimulus (p value < 1e-8), as in^10^. Our feature selection approach has two advantages: (1) voxels selected by our approach accurately capture brain response to visual input, and (2) the feature-set is compact, thereby ensuring time-efficient training.

In addition to feature selection from fMRIs, we modify the video frames in the following manner: first, the frame-rate of video input is 24 Hz, whereas the repetition time (TR) of fMRI is 0.72s (or 1.4 Hz). So, we subsample the temporal dimension from 24 Hz to 1.4 Hz. Then, we subsample the spatial dimensions of the video from 720×1024 to 224×224 for reducing run time.

### Cluster structure of predicted and actual latent vectors is similar

In our first set of results, we demonstrate that the cluster structure of predicted latent vectors computed using fMRIs is similar to latent vectors computed from video stimulus. This means that distance between pairs of latent vectors output by the encoder acting on input video frames is similar to distance between latent vectors computed using the non-linear maps acting on corresponding fMRI frames. This result shows that the output of the map acting on fMRI frames can serve as good approximations to output of the encoder acting on corresponding video frames, thereby allowing us to make predictions about video stimuli (i.e., video frames that the autoencoder has not seen during training).

We set up the experiment as follows: first, we divide the video inputs by keeping fifteen clips for training and one for testing. Then, we train the autoencoder model on the train set, holding out a clip from the train set in each iteration of cross-validation. After training, we input the train set into the encoder to obtain the (actual) latent representation. We then train the map from fMRI of a subject to latent representation. We use the fMRI frames corresponding to the video frames in the train set to train the non-linear map. We perform the training with cross-validation as before. This setup gives us three sets of latent vectors from both the encoder and the non-linear map. *First*, we have vectors corresponding to train set within each iteration of cross-validation (which both the encoder and non-linear map have previously seen). *Second*, we have the vectors in the hold-out set in the training procedure of the non-linear map (which the encoder has seen, but the decoder has not been trained on in the current iteration). *Third*, we have the vectors corresponding to the test video and corresponding test fMRI (which neither the encoder nor the fMRI have previously encountered). In each case, we cluster the outputs of encoder and non-linear map separately using k-means for k={3,4,…,20}. Then, we compute the adjusted rand index (ARI) between the clustering obtained. We repeat this entire process by holding each of the sixteen clips back for test on a randomly selected cohort of 10 subjects. The overall architecture is shown in Figure 4.

**Figure 5:**
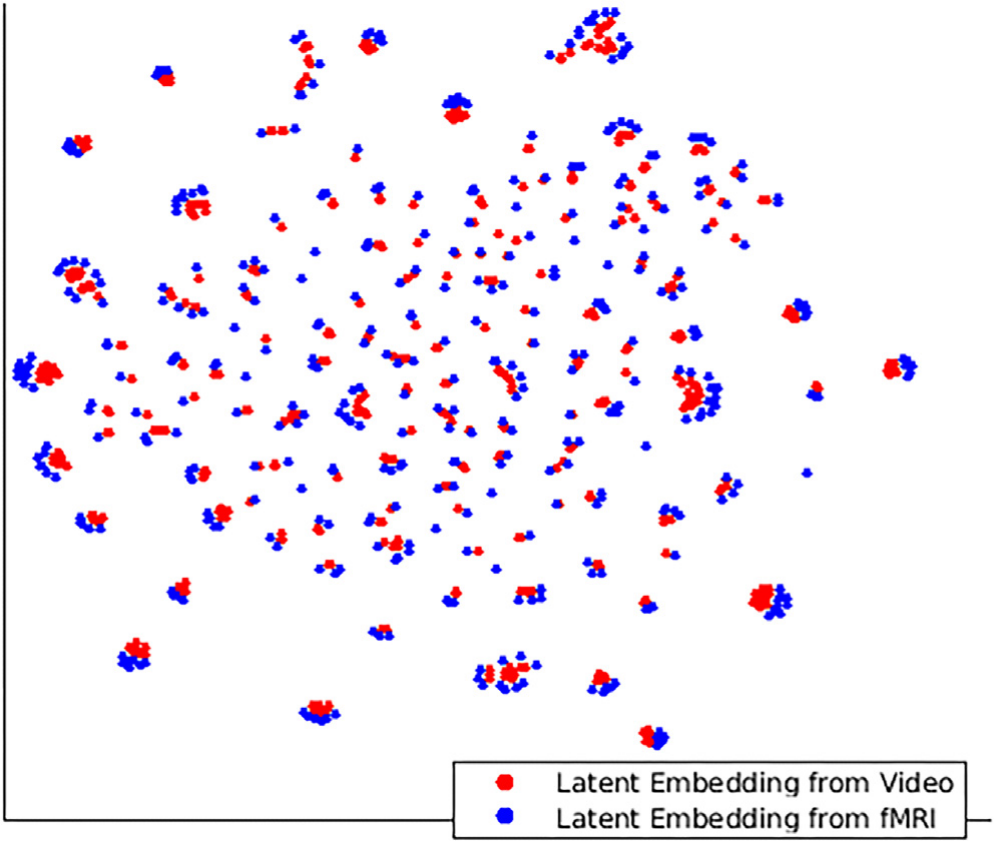
Visualization of latent representations computed from video frames processed using a trained encoder (blue) and from corresponding fMRI frames processed using a trained non-linear map (red) for a sample subject, after reducing to two dimensions using t-SNE For effective visualization, we have not explicitly numbered each frame.

**Figure 6:**
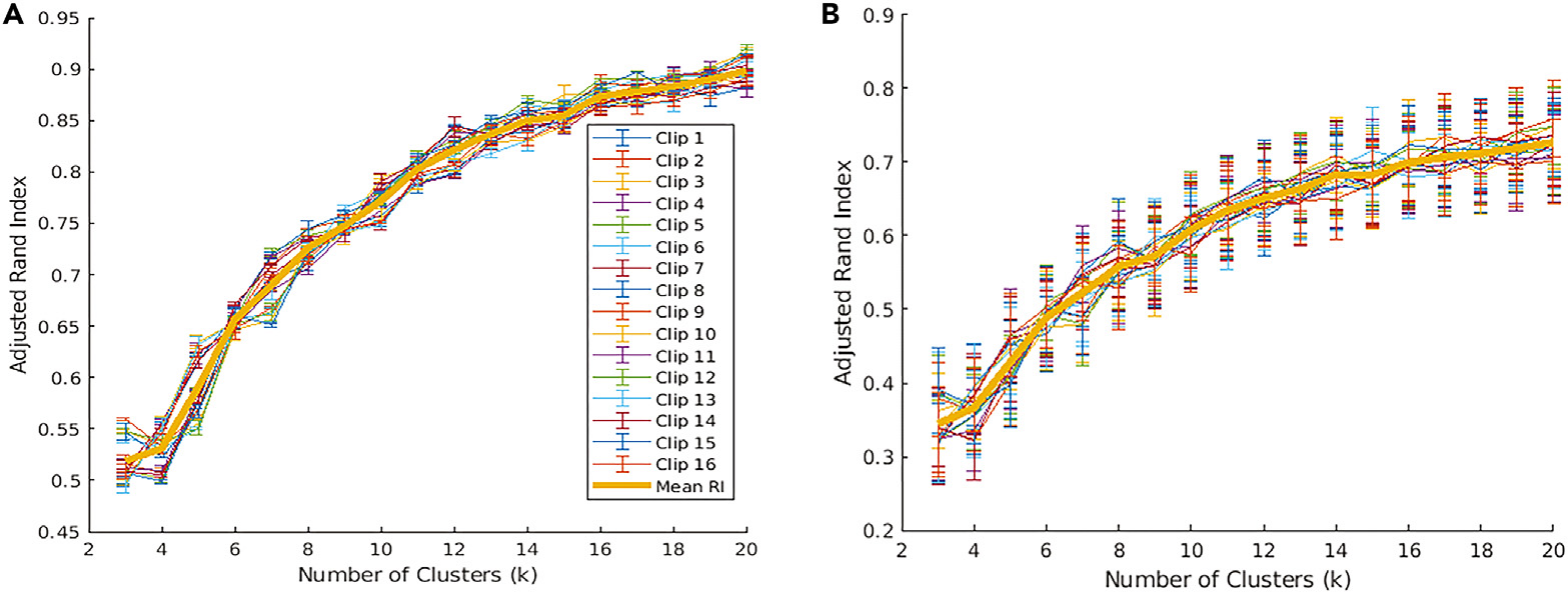
Plots show similarity of clustering in latent space of embeddings obtained from video frames processed using a trained encoder and functional MRI frames through a trained non-linear map (A) The relationship between cluster groupings and adjusted rand-index (ARI) when the encoder has been previously trained on the video frames. (B) The relationship between ARI and clustering when neither neural network has previously seen the video or corresponding fMRI frame. The plots are shown for different numbers of clusters (*k*). The high values of both measures show that embeddings obtained from the two routes are similar, thereby showing that the fMRI processed using the trained map can be used to approximate the video frames and encoder. Finally, we note that the measures plateau for larger values of *k*.

In the first case, where both encoder and non-linear map have previously seen the video and fMRI frames, respectively, we observe that the cluster structure of actual and predicted latent vectors are similar, as evidenced by the high ARI >0.8 for all k>5 across the entire cohort of subjects. Further, we also computed the k-nearest latent vectors (obtained from video frames) for every predicted latent vector (obtained from an fMRI frame). We report that in 68.2±4.7% of all time points across the cohort of subjects, we predict the correct stimulus by simply matching the closest latent vector (i.e., k-NN with k=1) obtained from a video frame to every latent vector obtained from the fMRI frame. The prediction goes up to 92±4.8% if we are allowed to correctly match to one of the three closest neighbors (i.e., k-NN with k=3). We visualize the clustering using the non-linear two-dimension embedding obtained from t-SNE^16^ for a typical subject in Figure 5. We can see that each cluster of points in the figure contains latent vectors from video and fMRI (blue and red, respectively). The ARIs obtained for the latter two conditions are presented in Figures 6A and 6B. We note that the high degree of agreement in cluster structure of the two latent representations across a range of cluster values supports our claim that fMRIs can be used to approximate visual stimulus in the latent space. Finally, Figure 6B shows that the ARI for k=10 is 0.70±0.03 when we used GRUs for the fMRI-Latent map.

We replaced our architecture with a purely CNN-based architecture.^9^ In this case, we observed that the ARI drops to 0.38±0.12 for k=10. A t test on the ARIs obtained by the two methods gives us a p value of <1e−40, confirming the superiority of our approach across choices of *k*. In another experiment, we replaced the latent vectors output by our encoders with motion energy features^17^ corresponding input video frames and fMRI-Latent Map (M) with these features as output. In this case, we observed ARI of 0.46±0.07. We summarize these results in row 1 of Table 1.

**Table 1:** Summary of the performance of related methods to demonstrate the significance of our results

Task	^9^	^17^	^10^	^11^	our method
ARI	0.38±0.12	0.46±0.07	xx	xx	0.70±0.03
Obj. predict. (Top 1)	28.1±10.8%	33.7±6.1%	xx	xx	50.9±13.7%
Obj. predict. (Top 5)	54.8±9.4%	49.5±4.3%	xx	xx	74.2±8.8%
Face detection	57%	57%	44%	36%	70%

The first row shows the adjusted rand index between the clustering obtained by actual and predicted latent vectors. The high ARI obtained by our approach shows that our predicted latent vectors are good approximation of actual latent vectors. The second and third row demonstrate that our approach outperforms other methods in being able to predict objects in visual stimuli. The fourth row shows that our approach is excellent in detecting when a subject is looking at a face. We use xx when methods are not applicable for specific results.

### Predicted latent vectors from fMRI encode semantic aspects of corresponding video frames

We now show that the predicted low-dimension latent embedding obtained from fMRIs encode information pertaining to objects found in the corresponding video frames. We show that our approach can be used to predict objects in the video frames, even when the autoencoder has not been trained on them. For the results we show in this section, we use an external, pretrained neural network, VGG16 on ImageNet database^6^ (a deep neural network pretrained on 14 million high-definition images to predict 22000 different categories). Specifically, we use weights from the VGG16-trained neural network available on Keras, which returns 1,000 categories.

**Figure 7:**
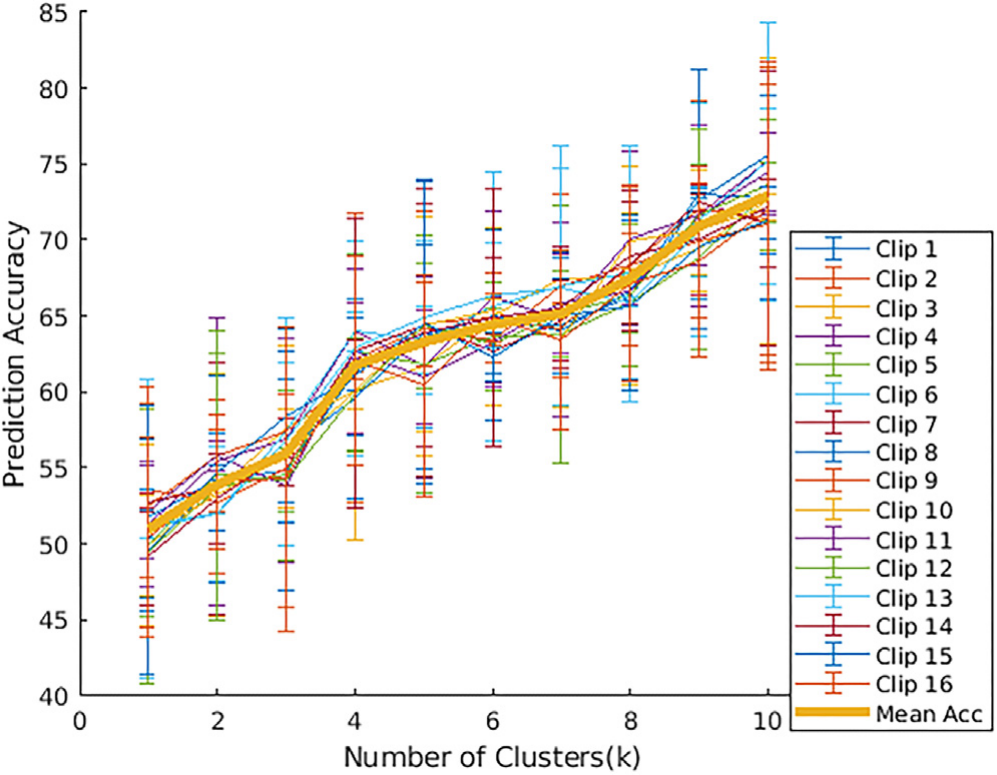
Plot shows the prediction accuracy when matching objects/classes recognized in visual stimulus (in latent space) and its corresponding *k*-closest neighbors among all train video frames (in latent space) We see that the prediction accuracy is >70% when we allow the fMRI frame to be matched to one of its ten closest Euclidean neighbors in latent space. We use VGG16 on ImageNet database to recognize entities in images.

To demonstrate this result, we train the autoencoder on the train video clips as before. Then, we train a map from the corresponding fMRI frames to the latent space vectors computed using the encoder on the video stimulus (the train set). We use this to predict the latent vectors on the test set. We note that the autoencoder has not previously seen the associated (test) video frames. We assign to each predicted latent vector its closest Euclidean neighbor among all vectors obtained from the train video frames. We then input the test video frame and the corresponding predicted video frame into VGG16 on ImageNet database to predict objects in each of them. We restrict ourselves to the top entry of the video frame corresponding to the fMRI (i.e., the object/class with the highest associated probability) and the top-*k* objects predicted in the video frame from the train set. The result is shown in Figure 7. We find a 50.9±13.7% overlap in the top (one) class and a 74.2±8.8% overlap between the top five classes predicted for all pairs of objects. These results were averaged over 16 different test clips and a random cohort of 10 subjects.

**Figure 8:**
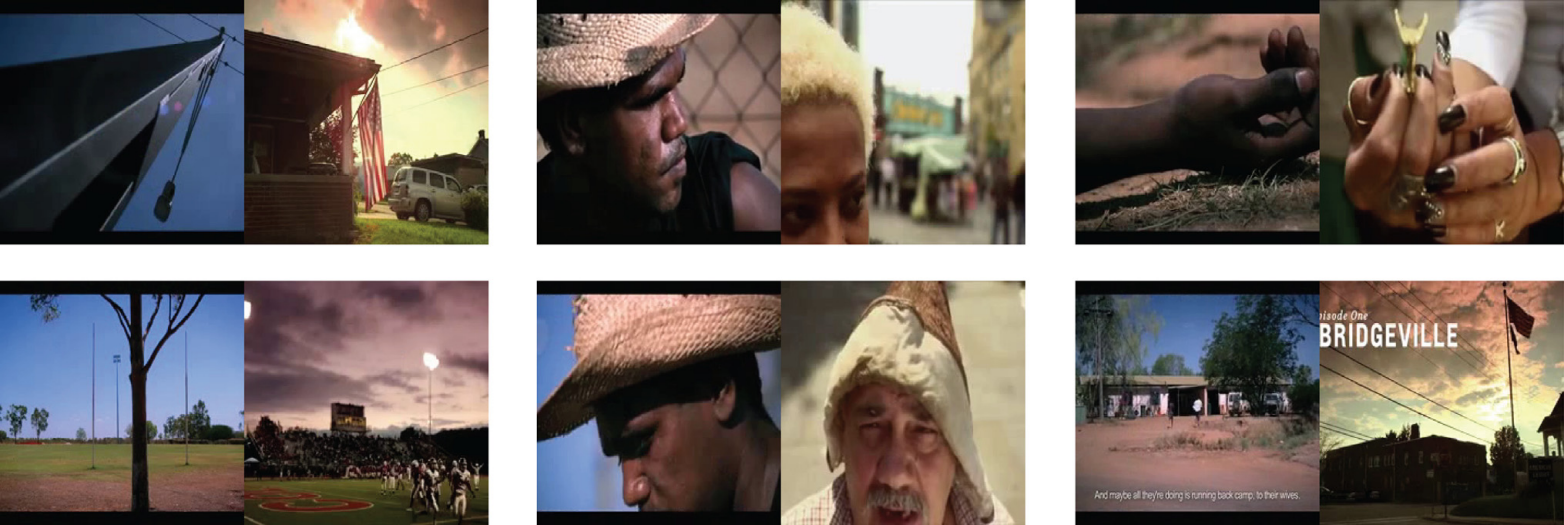
Examples of similarity of actual visual stimulus obtained by the clustering in latent space The left frame in each of these examples is computed using latent vectors from visual stimulus, whereas the right frame is its closest Euclidean neighbor among latent vectors computed from fMRI frames in the training set. In each of the images, we can see similarities in objects/faces in the background or foreground.

In Figure 8, we show examples to further highlight our point. In each of the instances shown, the image on the left is the visual stimulus corresponding to an fMRI frame from the test set; the image on the right is a video frame from the train set whose latent embedding is closest to the predicted latent embedding of the aforementioned fMRI frame. In each of the example pairs, we can see similarities. For instance, in the first pair, each image shows a building with the sky in the background. Similarly, the third pair shows images with hands where fingers are curled. We note that these images provide qualitative evidence to support our claim; however, the reasons behind some predictions are not always evident.

**Figure 9:**
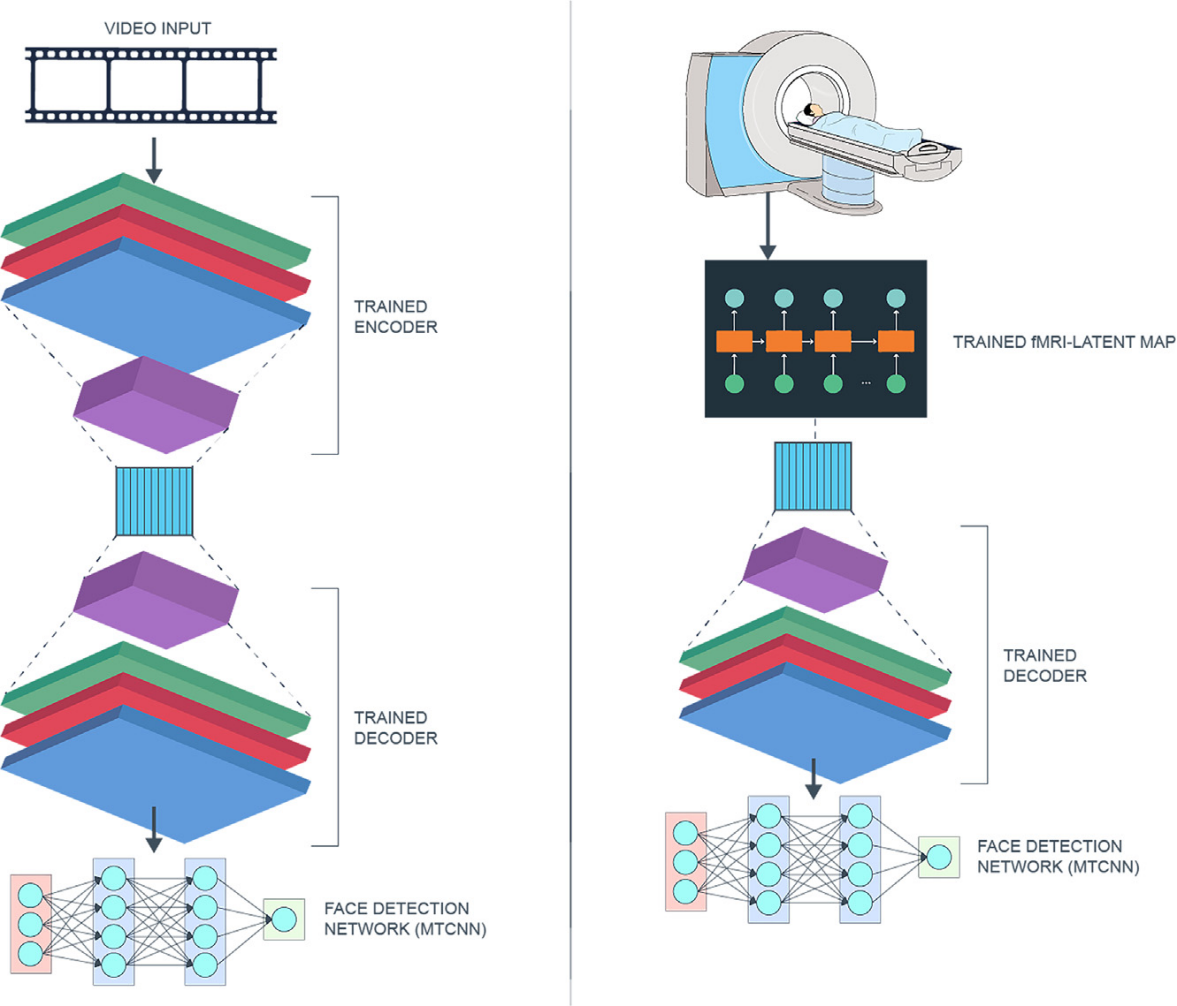
Schematic representation of face detection After training the autoencoder and the fMRI-Latent map, we detect faces as follows. First, test videos are passed through the autoencoder, and the output of the decoder is processed using MTCNN, a pretrained network to detect faces. This output serves as the ground truth. Then, we pass the corresponding fMRI frames into the map and the resulting latent vector through the decoder. The output of this is fed into MTCNN to give our prediction of face/no-face. Comparing the labels of ground truth and our prediction, we assess the performance of our framework.

**Figure 10:**
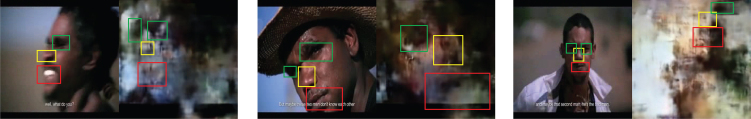
Examples of reconstructed faces In each example, the image on the left is the original image, and the image on the right is reconstructed from the corresponding fMRI frame. We input test fMRI frames into the non-linear map to obtain predicted latent vectors. Then, we input these vectors into the previously trained decoder. The bounding boxes represent the predicted eyes (green), nose (yellow), and mouth (red) by MTCNN.

We performed the same experiment with the CNN-based architecture proposed by Wen et al.^9^ and found 28.1±10.8% overlap in the top (one) class and a 54.8±9.4% overlap between the top five classes predicted for all pairs of objects. In the case of Nishimoto et al.,^17^ we found 33.7±6.1% overlap with the top class and 49.5±4.3% overlap with one of the top five classes. These results are summarized in rows 2 and 3 of Table 1.

### Object detection and reconstruction

In this final result, we reconstruct visual stimulus from fMRI. As before, we train the autoencoder on train set of video clips, followed by training the non-linear map on corresponding fMRI frames. This is followed by inputting the test video frames into the autoencoder to get a set of reconstructed images. Finally, we input the test fMRI frames into the non-linear map to get predicted latent vectors and pass these vectors through the previously trained decoder to get another set of reconstructed images. The overall architecture is in Figure 9. Unlike previous experiments where we made predictions using original images, here our predictions are based wholly on reconstructed images. Because reconstructed images are from the test set, the images are not sharp enough to use multi-class classifiers such as VGG16. We limit ourselves to detect the presence or absence of an entity that appears often in images—faces. In Figure 10, we show some examples. In each of these, the image on the left is the original image, and the image on the right is the reconstructed image.

**Figure 11:**
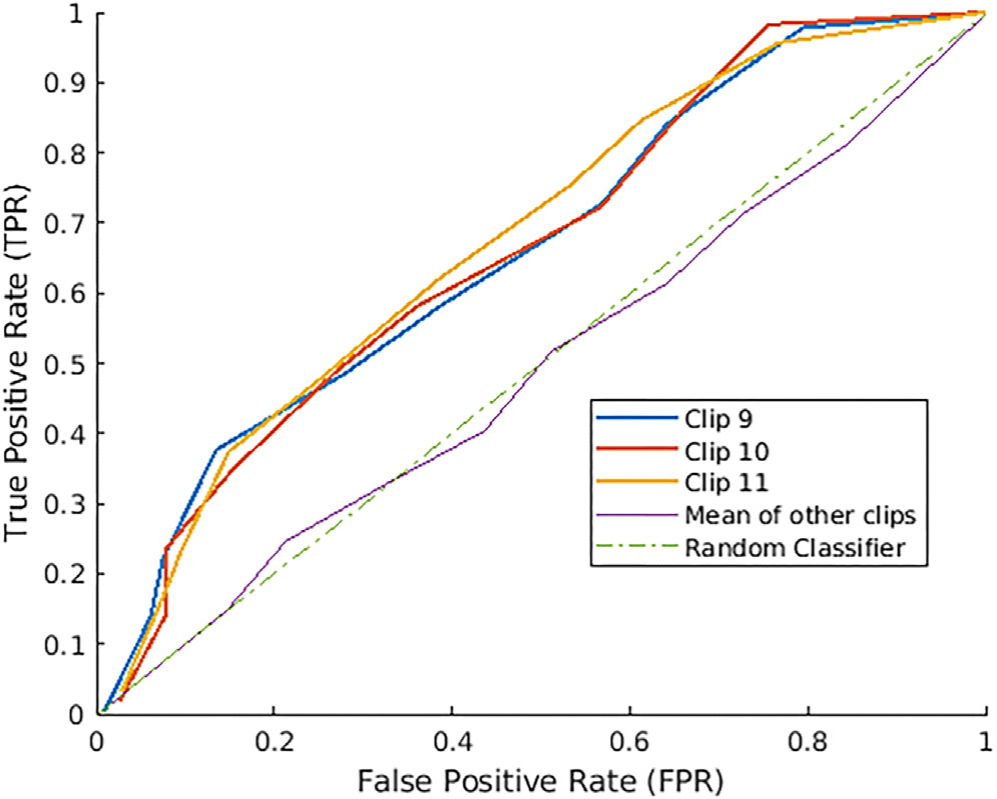
Receiver operating characteristic (ROC) obtained by varying confidence thresholds for the three stages of MTCNN We note the excellent performance of our framework for scenes with significant number of faces.

We show that the output of our framework can be used to detect the presence of faces in individual frames in an automated manner. First, we input both sets of reconstructed images—output of the autoencoder and output of non-linear map to decoder into the pretrained Multitask Cascading Neural Networks (MTCNN),^8^ available as an open-source project with MIT license. The network outputs “face” or “no face” for each input image. MTCNN proceeds in three stages. In the first stage, it finds candidate windows (bounding boxes within which faces are present) using a shallow CNN. Then, it refines the windows to reject a large number of non-face windows using a deeper CNN. Finally, it uses a third (deep) CNN to output facial landmarks (left and right eyes, nose, mouth). Very high thresholds lead to missed faces (false negatives), whereas very low thresholds lead to misidentified faces (false positives). In a grid search, we vary the confidence thresholds in MTCNN for each of the three stages and plot the resulting Receiver Operating Characteristic curve (False Positive Rate vs. True Positive Rate) in Figure 11. We find that threshold values of [0.5,0.6,0.6] work well for our setup. We restrict ourselves to input scenes where faces appear often (Clips 9, 10, 11). We observe that our framework performs very well in terms of predicting the presence of faces in frames with impressive accuracy, considering the difference in temporal and spatial resolution between video input and fMRI scans. Using GRUs in the fMRI-Latent map, we obtain >70% accuracy in correctly detecting faces, which drops to 57% when we use the CNN-based architecture proposed by Wen et al.^9^ Other methods designed specifically on face detection, such as VanRullen and Reddy^10^ and Cowen et al.,^11^ yield prediction accuracies of 44% and 36%. We note that the performance of the latter two methods suffers because they are designed to handle still frames of cropped faces, captured from the front (like passport photographs), as opposed to video data that consistently have moving frames, with faces captured at different angles, and with various objects in the background. The face detection accuracy in the case of Nishimoto et al.^17^ was 57%. In the reconstruction stage, their method averages the images corresponding to the five closest motion energy vectors (analogous to our latent vectors). We find that this procedure of “hardcoding” the decoder produces poorer reconstructions. Instead, our approach is completely data-driven, with the video frames themselves guiding the weights of the decoder.

In clips with few frames with faces, we observe that our approach does not perform well. We posit that there are three reasons for this: (1) the pre-trained network MTCNN gives false positives, even when images are of good resolution, which leads to many “face” predictions even when there are no faces; (2) there is temporal persistence in neuronal activity in the seconds following a scene with a face; and (3) our decoder is not optimized to reconstruct faces in particular. Although these considerations are significant, the purpose of our experiment is to demonstrate the feasibility of using either human annotators or better face detection methods to reconstruct faces from fMRIs with high accuracy.

#### System specification

The results discussed in this section were obtained on an Nvidia Tesla P100-PCIE GPU with 16 GB RAM. We used Keras 2.4.0 with Tensorflow 2.4.1 on Python 3.7. Both neural networks used Adam optimizer, with learning rate set at 0.001, first-order exponential decay $\beta_{1}=0.99$, and second-order term $\beta_{2}=0.999$. For numerical stability $\hat{\epsilon }=1e-7$.^18^ The total emissions for all our experiments are estimated to be 108 kgCO2eq. Estimations were conducted using the Machine Learning Impact calculator (https://mlco2.github.io/impact#compute) by Kingma and Ba.^19^

## Discussion

Our work demonstrated the feasibility of reconstructing images from observed fMRI responses. In this work, the optimization functions for the encoder-decoder (L) and the non-linear map (L′) were done in a sequential manner. However, an approach to jointly optimize the two functions would give (1) an encoder that attempts to take the fMRI map’s objective into account and (2) a decoder that is aware of latent vectors computed from fMRIs. This can result in better reconstructions. Second, we made the decision of subsampling videos to match the sample rate of fMRIs; however, it may be better to find more effective ways to reduce temporal resolution of videos, without explicitly excluding a majority of the frames. This is particularly important because although fMRIs are temporally of lower resolution, the values themselves are functions of all the visual frames seen. Third, we note that we have excluded audio in this study. Finally, we note that our model is trained and tested on the same subject. All of these provide avenues for continuing research.

Our motivation for this work is to understand the processes that underlie audiovisual perception in human brains. Our methods can be applied to other time-series modalities, such as EEGs, as part of a brain-computer interface that can assist people with strokes and other neurological disorders. Studies on disregulation of these cognitive processes can also inform treatment strategies.

### Related literature

#### Reconstructing natural images

In a landmark paper, Miyawaki et al.^20^ reconstruct visual images from fMRI frames using a combination of Multiscale Local Image Decoders. In their approach, contrast-defined 10×10 patch images are presented to subjects. Then, linear weighted multivoxel fMRI signals are used to predict contrasts of local image bases. The local image bases are then multiplied by predicted contrasts to reconstruct the image. Although their simple linear model yields impressive results, we note that their approach works only for controlled inputs and not for naturalistic inputs. Beliy et al.^21^ propose a method for image reconstruction from fMRIs when labeled pairs of (image, fMRI) are not available. Their method uses two networks: (1) image to fMRI and (2) fMRI to image. Concatenating the two back-to-back allows for training with both types of unlabeled data. Mozafari et al.^22^ propose a method based on a large-scale bidirectional generative adversarial network (BigBiGAN) to decode and reconstruct natural scenes from fMRI patterns. In a related effort, Huang et al.^23^ present a GAN-based latent-space encoder and latent-space decoder to reconstruct natural scenes. Shen et al.^24^ present a method where pixel values of an image are optimized to make its deep neural network features (at different levels) similar to those decoded from fMRI. They show that this approach provides reliable reconstruction of natural images. In a recent survey paper, Huang et al.^25^ compare different approaches in reconstruction of natural scenes. Date et al.^26^ show that electrocorticography signals can also be used to reconstruct natural scenes. *In this paper, we handle data that are significantly more complex than prior results—each frame is related to frames that precede and follow it, which is not the case with static images used in these prior results. This confounds reconstruction because of inertia in neural response. Furthermore, video frames have moving objects, leading to significant degradation of image frames extracted due to blurring and motion effects.*

#### Reconstructing faces

VanRullen and Reddy^10^ and Lin et al.^27^ propose GANs in conjunction with linear maps and CNNs, respectively, to reconstruct faces from fMRI. Our approach is similar to^10^ in the sense that they also build a map between fMRI and latent features. However, our model works well in practice for reconstructing from generic moving images (or video), whereas their method works only on images. In an earlier result, Cowen et al.^11^ propose an approach for learning a function between PCA-based components obtained from images of faces (called eigen-faces) and their corresponding fMRI response. In our approach, we do not restrict the domain of inputs to high-quality images but we can still reconstruct faces. Hence, our approach is more flexible and can accommodate a wide variety of visual inputs. Indeed, these prior results show that face reconstruction from fMRI is possible. However, it is important to note that in other methods (1) the dataset is wholly composed of high-quality images of faces; (2) viewed from the front, with very little or no background; and (3) each image is viewed for a few seconds. In our reconstruction results, faces are reconstructed from a fMRI dataset in which (1) visual input contains several frames with no faces and () people are moving (hence, poses are highly dynamic, and each pose is seen for a fraction of a second, as opposed to several seconds). Hence, our reconstructions are novel and noteworthy.

#### Reconstructing video frames

Wen et al.^9^ propose a deep learning approach to model a bidirectional relationship between video stimuli and fMRI. However, their approach uses only CNNs and therefore does not encode memory in any way. Nishimoto et al.^17^ describe a method to encode image data using Gabor Filters and predefined hemodynamic response filters, which results in “Motion Energy Features” for each input frame. Then, they fit voxel-wise models to these features. To reconstruct, they predict the feature vectors for a test fMRI frame and simply average the top five maximum aposteriori vectors from the train set. We show significantly better results for our models compared with both methods. Furthermore, our approach is more natural in that we exploit memory, much like the human brain itself. Furthermore, as our method does not require hard coding of the model, we let the data drive the decoding phase. Our approach can be easily integrated into applications, including brain-computer interfaces, augmenting AR/VR devices, and behavioral studies for neurodegenerency.

### Limitations of the study

In this study, we present results on the Human Connectome Project, a large-scale, well-accessed, and standard dataset. This is largely because HCP data have excellent spatial and temporal resolution. Further studies to characterize performance on other datasets with poorer resolution would be very useful. Furthermore, this study is limited to young and healthy adults. Neurodegenerative diseases related with cognitive decline could change the mechanisms by which video inputs are processed.

## STAR★Methods

### Key resources table

**Table:** 

REAGENT or RESOURCE	SOURCE	IDENTIFIER
**Deposited data**		

HCP Dataset	Human Connectome Project Consortium	https://db.humanconnectome.org/

**Software and algorithms**		

Code	Authors	https://doi.org/10.13140/RG.2.2.10457.49763

### Resource availability

#### Lead contact

Further information for resources should be directed to Vikram Ravindra (vikram.ravindra@uc.edu).

#### Materials availability

This study did not generate new unique reagents.

#### Data and code availability

• This paper uses the publicly available open-source Human Connectome Project Dataset. The DOI is available in the key resources table.
• Code can be obtained at https://doi.org/10.13140/RG.2.2.10457.49763.
• Any additional information required to reanalyze the data reported in this paper is available from the lead contact upon request.

### Method details

Let V be an ordered set of video frames and F be the corresponding ordered set of fMRI frames. For each $v_{i}\in V$, we can define $f_{i}\in F$ as $f_{i}=F\left(v_{i}\right)$, where *i* corresponds to a time-point and $F:R^{l\times w}\to R^{s}$ is a function that maps a video frame of length *l* and width *w* to neuronal activity of *s* voxels. We note that each voxel is in three spatial dimensions; however, we have flatten the 3D fMRI image matrix for convenience. Our goal is to learn a map from the fMRI space to video space. We also note that temporal resolution of video frames is much higher than corresponding fMRI frames (≈ 41 ms/frame for videos and ≈ 0.7s/frame for fMRI). We handle this mismatch by subsampling video frames.

#### Constructing a common latent space for video and fMRI features

To construct a map from fMRI to video space, we first express both video frames and corresponding fMRI frames in a common latent space. We map video frames to a latent space and reconstruct the image from the latent space using an encoder(E)-decoder(D) architecture (also known as autoencoder). We define E as the ordered set of vectors that are produced when video frames in V are input to the encoder and denote each vector in E as $e_{i}=E\left(v_{i}\right)$. In other words, we train the neural networks so as to minimize the following:

(Equation 1) $$\min_{E,D}\sum_{i\in \left\{1,\ldots ,t\right\}}L\left[v_{i},D\left(e_{i}\right)\right]\text{,}$$

where L is a suitably defined loss function.

Once the autoencoder is trained, we map fMRI frames to the same latent space using a non-linear map **M**. The objective function for this map minimizes the loss between the mapped fMRI frame and the latent representation of the corresponding video frame. It is given by:

(Equation 2) $$\min_{M}\sum_{i\in \left\{1,\ldots ,t\right\}}L^{\prime }\left[e_{i},M\left(f_{i}\right)\right]$$

where $L^{\prime }$ is (another) loss function. In our implementation, we used mean squared error for L and $L^{\prime }$ with Adam optimizer.^18^ Stated otherwise, the latent representation (output) of the map of an fMRI image should best match the latent representation from the encoder applied to the corresponding video frame.

This setup creates a common space within which we can express both video frames and fMRI frames. Hence, there are two pathways to a reconstructed video frame: (i) video space → latent space → video space; and (ii) fMRI space → latent space → video space. A schematic representation of this procedure is shown in Figure 1. The solid arrows (1 and 3) correspond to the training of the two neural networks, whereas the dotted arrows (2 and 4) denote steps where video and fMRI frames are input into previously trained neural networks. The training procedure is as follows: first, we partition the video and fMRI dataset into train and test by holding back one (of sixteen) video clips for test. Using the remaining clips, we train using leave-one-out cross-validation. We then generate the latent vectors from all train video frames. Using the corresponding fMRI frames, we train the fMRI-Latent map. Note that we do not use the test video frames or their corresponding fMRI frames in any manner while training. In the rest of this section, we describe the architectures of the neural networks and feature selection procedure.

#### Autoencoder framework

The input to our encoder is a series of video frames (h,w,c,t), where *h* represents the height and *w* represents the width of the image frame, *c* represents the number of channels, which in our case is three colors (RGB), and *t* the time dimension. Unlike autoencoder architectures used to reconstruct simpler image inputs (such as static faces), our input has memory inherently encoded in the sequence of video frames. To exploit this structure in data, our encoder is a deep neural network comprising convoluted neural networks (CNNs) and recurrent neural networks (RNNs) implemented as Gated Recurrent Units (GRU), as described in Figure 2.

The input layer is followed by an alternating sequence of convoluted neural networks (CNNs) and pooling layers (using max pooling). In each CNN layer, we use Rectified Linear Unit (ReLU) as the activation function, followed by batch normalization. We use maxpool as the pooling technique to down-sample spatial dimensions of the matrix with pool size (2,2). Finally, the output of the CNNs is flattened and input into a recurrent neural network. We use stateful Gated Recurrent Units (GRU) by,^28^ and a hyperbolic tangent (tanh) as activation function for the GRU layer.

In our experimental results, the input to the encoder is a series of images, each of size 224×224×3. The outputs are latent representations in 1024 dimensions. We note that the CNN-RNN architecture that we use in our encoder is similar in spirit to.^29^ However, our objective is to reconstruct the image; i.e., the output is a vector, rather than scalar (predicted class).

Our decoder is more shallow than the encoder. First, we reshape the output of the encoder (i.e., latent vectors) and follow it with a sequence of up-sampling with step size of (4,4) and inverse convoluted layers (CNN transpose) to compute reconstructed video frames.

#### fMRI-latent map

The fMRI to Latent space map is a non-linear map from vectorized fMRI frames to their corresponding latent vectors. Unlike the approach of,^10^ where a linear map was sufficient to capture the relationship between fMRI and visual features, derived from a high-quality celebrity face dataset,^30^ we need to learn a more complex map for two reasons. First, our input is more complicated in terms of the variety of scenes it captures. Our dataset has natural scenes, thereby increasing the number of objects on screen, differences in lighting, camera angles, and other image attributes. Second, as both the video data and fMRI are time-dependent signals, a linear map between the matrices of fMRI response and latent vectors is insufficient to capture temporal relationships. For these reasons, we use a non-linear map with memory in our architecture. Our choice for GRUs in this learnt map is motivated by the fact that a frame of video input (albeit downsampled) contains useful information about the next frame(s). We ensure that the latent embedding encodes this temporal dependence because successive fMRI frames also have corresponding temporal dependence, something that a non-linear memoryless framework like CNN cannot achieve. Furthermore, fMRI frames (unlike video) are very noisy. Hence, guiding the map toward a predicted latent vector (from the map) that is “close” to the predicted latent vector of the previous step is desirable. We implement it as two layers of stateful GRUs using tanh function for activation, and a dropout rate of 0.1. In Sections cluster structure of predicted and actual latent vectors are similar and object detection and reconstruction, we demonstrate significant improvement in performance of our RNN-based architecture over conventional CNN-based architectures.

We find empirically that adding more layers of GRU leads to overfitting to the train set, thereby yielding poor test accuracy. The map is summarized in Figure 3. We provide additional details of number of training samples, rates, etc., in our experimental results.
